# Depletion of Trichoplein (TpMs) Causes Chromosome Mis-Segregation, DNA Damage and Chromosome Instability in Cancer Cells

**DOI:** 10.3390/cancers12040993

**Published:** 2020-04-17

**Authors:** Angela Lauriola, Andrea Martello, Sebastian Fantini, Gaetano Marverti, Tommaso Zanocco-Marani, Pierpaola Davalli, Daniele Guardavaccaro, Sabine Mai, Andrea Caporali, Domenico D’Arca

**Affiliations:** 1 Department of Biomedical, Metabolic and Neural Sciences, Via G. Campi 287, University of Modena and Reggio Emilia, 41125 Modena, Italy; angela.lauriola@univr.it (A.L.); gaetano.marverti@unimore.it (G.M.); pierpaola.davalli@unimore.it (P.D.); 2 Department of Biotechnology, University of Verona, 37134 Verona, Italy; daniele.guardavaccaro@univr.it; 3 University/British Heart Foundation Centre for Cardiovascular Science, The Queen’s Medical Research Institute, University of Edinburgh, Edinburgh EH16 4TJ, UK; a.martello@ucl.ac.uk; 4 UCL Institute of Ophthalmology London, London EC1V 9EL, UK; 5 Department of Life Sciences, Via G. Campi 213/d, University of Modena and Reggio Emilia, 41125 Modena, Italy; sebastian.fantini@unimore.it (S.F.); tommaso.zanoccomarani@unimore.it (T.Z.-M.); 6 Cell Biology, Research Institute of Oncology and Hematology, University of Manitoba, CancerCare Manitoba, Winnipeg, MB R3E 0V9, Canada; sabine.mai@umanitoba.ca

**Keywords:** mitostatin, cancer, spindle assembly checkpoint (SAC), Mad2, chromosome mis-segregation, chromosome instability (CIN), DNA damage, spectral karyotyping (SKY)

## Abstract

Mitotic perturbations frequently lead to chromosome mis-segregation that generates genome instability, thereby triggering tumor onset and/or progression. Error-free mitosis depends on fidelity-monitoring systems that ensure the temporal and spatial coordination of chromosome segregation. Recent investigations are focused on mitotic DNA damage response (DDR) and chromosome mis-segregations with the aim of developing more efficient anti-cancer therapies. We previously demonstrated that trichoplein keratin filament binding protein (TpMs) exhibits hallmarks of a tumor suppressor gene in cancer-derived cells and human tumors. Here, we show that silencing of TpMs expression results in chromosome mis-segregation, DNA damage and chromosomal instability. TpMs interacts with Mad2, and TpMs depletion results in decreased levels of Mad2 and Cyclin B1 proteins. All the genetic alterations observed are consistent with both defective activation of the spindle assembly checkpoint and mitotic progression. Thus, low levels of TpMs found in certain human tumors may contribute to cellular transformation by promoting genomic instability.

## 1. Introduction

Genome integrity is continuously threatened by DNA lesions resulting from both endogenous and exogenous stress that causes genome damage and, consequently, genomic instability. Eukaryotic cells are endowed with interrelated pathways that face DNA damage, allowing DNA repair and correct cell cycle progression. This defensive system is comprehensively known as DNA damage response (DDR). DNA repair and cell cycle control are deeply related, as the correct duplication and transmission of genetic material are the main objectives of the cell division process [1]. The proper conditions for cell division are set during the cell cycle thanks to the action of multiple checkpoints. Increasing evidence reveals that errors in mitosis promote the direct and indirect acquisition of DNA damage and chromosome breaks that trigger DDR during mitosis (mitotic DDR) [2]. How various types of mitosis-linked DNA damage engage DNA damage signaling pathways and repair components is unknown [3,4]. In recent years, a novel interplay between mitotic DDR and spindle assembly checkpoint (SAC) during mitosis has been highlighted [1,5]. Even if the SAC mechanism is not fully understood, the SAC satisfaction results as an accurate step to guarantee high fidelity in DNA replication during mitosis and, consequently, for maintaining genome stability. The mitotic spindle can control the interaction between microtubules and chromosome kinetochore, thus allowing the separation of chromatids and segregation of chromosomes into two new cells. Chromosomes have to be properly bi-oriented, as proper chromosome orientation during mitosis enables normal mitotic segregation so that unequal segregation (mis-segregation) and an imbalanced set of chromosomes in the new cells (i.e., aneuploidy progeny) are prevented. Mis-oriented and aberrant mitotic spindle are generally followed by anomalous SAC activity and, finally, by numerical and structural chromosome aberrations. When chromosome kinetochore is unattached or weakly attached to microtubules, SAC results unsatisfied and activates a network able to arrest cell cycle, delay cells undergoing mitosis and/or exit from mitosis that ultimately delays cell division. SAC, as all checkpoints, does not permanently block cell cycling. As soon as kinetochores are stably attached to the spindle, SAC is inactivated and the cell cycle block is removed, so that chromosome segregation and cell division proceed. Genomic instability referring to increased genome alterations during cell cycle is frequently detected in human cancers to be considered a tumor hallmark [6]. Genomic instability leads cancer cells in different tissues of different organs to acquire functional capabilities that allow them to survive, proliferate and disseminate. Typical features of many types of human cancers are incorrect chromosome segregation and somatic copy number alterations, i.e., aneuploidy. Definite types of aneuploidy that likely act through different mechanisms have been proposed to drive tumorigenesis or predict the occurrence of hallmarks of cancer, such as cell proliferation and immune evasion [7]. Trichoplein/Mitostatin, trichoplein keratin filament binding protein (TpMs, TCHP), is a cytoplasmic protein that binds keratin filaments and desmosomes in well-differentiated cells [8]. TpMs regulates also endoplasmic reticulum–mitochondria juxtaposition [9]. TpMs partly localizes to the mitochondria, being involved in the morphological and ultrastructural organization of the organelle [10], and is also involved in the mitophagy process that is evoked by the decorin protein [11]. Trichoplein links autophagy with endothelial cell functions [12]. TpMs appears evolutionarily conserved among several species and ubiquitously expressed in many human tissues, although at different levels. We previously cloned and characterized TpMs through genetic and functional studies demonstrating that TpMs exhibits tumor suppressor activity in vitro and in vivo. Ectopic expression of TpMs in cancer-derived cell lines inhibits cell growth as well as cell migration, invasion, adhesion in vitro, and tumor formation in vivo. TpMs overexpression decreases the expression and phosphorylation of heat shock protein Hsp27 in cancer-derived cell lines, and also inhibits cell migration, while facilitates apoptosis. TpMs gene location on human chromosome 12q24.1 was previously identified as Ts12q for tumor suppressor at 12q [10,13]. We found different frequencies of gene mutation and different levels of TpMs downregulation in malignant neoplasms as advanced bladder cancer, breast carcinomas and prostate cancer in respect to their healthy counterpart [10,13]. Low TpMs levels in the cells are suggested to contribute to malignant transformation. Although intriguing, TpMs functions have yet to be defined. TpMs likely activates the mitotic kinase Aurora A through a direct molecular interaction during centrioles’ activation at G1-phase, thus negatively regulating primary cilia assembly in this phase [14]. In vitro experiments show that TpMs binds to Odf2 and ninein proteins, thereby controlling microtubule anchoring to centrosome [15]. Considering that functional defects of centrosomes and microtubule dynamics are associated with mitotic failure, we hypothesized that TpMs might play a role in proper cell cycle progression. Here, we show that TpMs silencing causes abnormal spindle assembly checkpoint (SAC). The latter event is known to weaken chromosome segregation during mitosis, and to associate with aberrant mitosis progression. We found that TpMs interacts with Mad2 protein, which is a key regulator of the SAC, while TpMs silencing associates with decreased Mad2 level. We also detected DNA damage after TpMs depletion together with numerical and structural chromosome aberrations. All these alterations are consistent with defective SAC activation that may affect the cell progression in mitosis. Investigations on TpMs role during mitosis might deepen the knowledge of normal as well as pathological cells growth, also considering that the decreased TpMs levels detected in biopsy specimens might be exploitable for selecting and stratifying cancer patients in different groups. The individual TpMs levels of patients might represent a novel marker to be included in standard molecular and clinical tools.

## 2. Results

### 2.1. TpMs Is Required for Proper Progression through Mitosis and Spindle Checkpoint Activation

**Figure 1:**
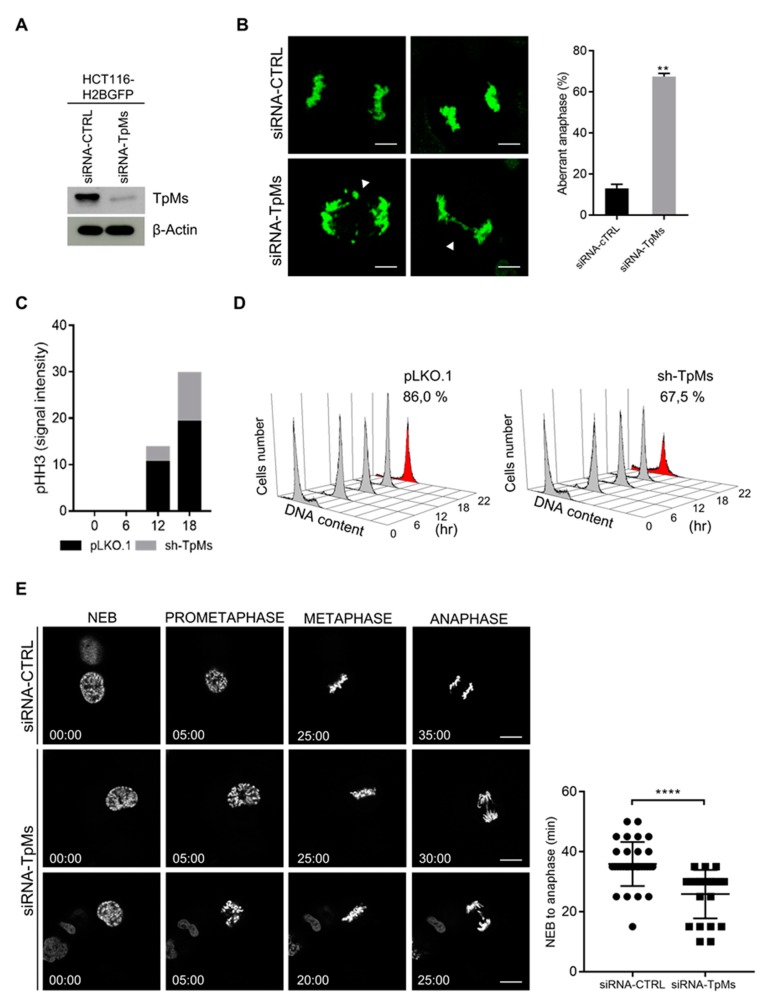
TpMs-depleted cells display chromosome segregation errors and defective spindle checkpoint. (**A**) Immunoblot showing decreased levels of TpMs after transient transfection with siRNA-CTRL and siRNA-TpMs in HCT116 cells stably expressing enhanced green fluorescent protein-labelled histone H2B. (**B**) Representative pictures of lagging chromosomes and chromosome bridges in anaphase (white arrowheads) (left panel, HCT116 cells) scale bars 10 µm. Quantification as percentage of aberrant anaphases (right panel) (*p*-value = 0.002). (**C**) Phosphorylation of histone H3 were assessed by immunoblotting and quantified by densitometry in TpMs-deficient HeLa cells. Cells were synchronized by aphidicolin treatment and released in nocodazole at different times. (**D**) Flow cytometry analysis of HeLa cells stably transfected with shRNA-TpMs vector or control vector (pLKO.1). Cells were released from a G1/S phase block (obtained by aphidicolin) into a nocodazole-containing medium and collected at the indicated times. (**E**) HCT116 were stably transfected with enhanced green fluorescent protein-labelled histone H2B and transiently transfected with siRNA-CTRL and siRNA-TpMs. The average time from nuclear envelope breakdown (NEB) to anaphase onset is measured by time-lapse microscopy. Each symbol in the scatter plot represents a single cell (right panel) (*p*-value < 0.0001). Representative fluorescence video-microscopy series of HCT116 cells (left panel). Scale bars 10 µm. Error bars represent s.d. (*n* = 3).

To investigate the role of TpMs in cell division, we silenced the expression of TpMs by RNA interference in human colon carcinoma HCT116 cells expressing enhanced green fluorescent protein-labelled histone H2B (Figure 1A, Figure S1). TpMs-depleted HCT116 cells (siRNA-TpMs) and control cells (siRNA-CTRL) were blocked at the G1/S phase transition by aphidicolin treatment and then released from the block to allow progression through mitosis. We observed an increased number of chromosome bridges and lagging chromosomes in TpMs-depleted cells in comparison with control cells (Figure 1B, left). Lagging chromosomes and chromosome bridges are typical hallmarks of chromosome mis-segregation, premature sister–chromatid separation and chromosomal end-to-end fusions. The chromosome aberrations observed in TpMs-depleted HCT116 cells were quantified and plotted as a histogram in Figure 1B, right. Further support for the role of TpMs in the spindle assembly checkpoint (SAC) comes from experiments in which cells were synchronized with an alternative technique (Figure S2A). The chromosome aberrations in non-transformed cell line Human Umbilical Vein Endothelial Cells (HUVEC) was also observed (Figure S2B). These findings suggest that the spindle assembly checkpoint (SAC), a surveillance mechanism that ensures the fidelity of chromosomal segregation during mitosis, might be defective in TpMs-depleted HCT116 cells. It has been shown that cells displaying defective SAC are generally prone to mitotic slippage in the presence of strong spindle checkpoint triggers such as nocodazole. To test whether TpMs-depleted cells may be prone to mitotic slippage, we evaluated the mitotic index of nocodazole-treated TpMs-depleted (sh-TpMs) cells. Cells were released from the G1/S block into a nocodazole-containing medium and collected at different time points. The levels of the mitotic marker phospho-Histone H3 (pHH3) were then assessed by immunoblotting and quantified by densitometry. As shown in Figure 1C, TpMs-depleted cells display a decreased pHH3 level when compared to control cells. The same results were obtained when mitotic progression was monitored by flow cytometric assay (Figure 1D, Figure S2C). These findings indicate a reduced mitotic index of TpMs-depleted cells despite the fact that cells progress through the S- and G2-phase and synchronously enter mitosis with normal kinetics (Figures S2C,D and S3). Altogether our results suggest that the silencing of TpMs expression results in a defective activation of the spindle mitotic checkpoint. To confirm these findings, we analyzed the progression of TpMs-depleted cells through mitosis by live-cell imaging. HCT116 cells expressing enhanced green fluorescent protein-labelled histone H2B were transduced with TpMs or control siRNA and analyzed by time-lapse microscopy (see Materials and Methods section). The average duration time (minutes) from nuclear envelope breakdown (NEB) to anaphase onset was decreased in TpMs-depleted HCT116 cells (siRNA-TpMs) in comparison to siRNA-CTRL (control cells) (Figure 1E, left, and Videos S1–3). We also observed different types of chromosome aberrations in anaphase such as lagging chromosomes and chromosome bridges, which were plotted as histograms (Figure S2E).

### 2.2. TpMs Depletion Leads to Chromosomal Instability

**Figure 2:**
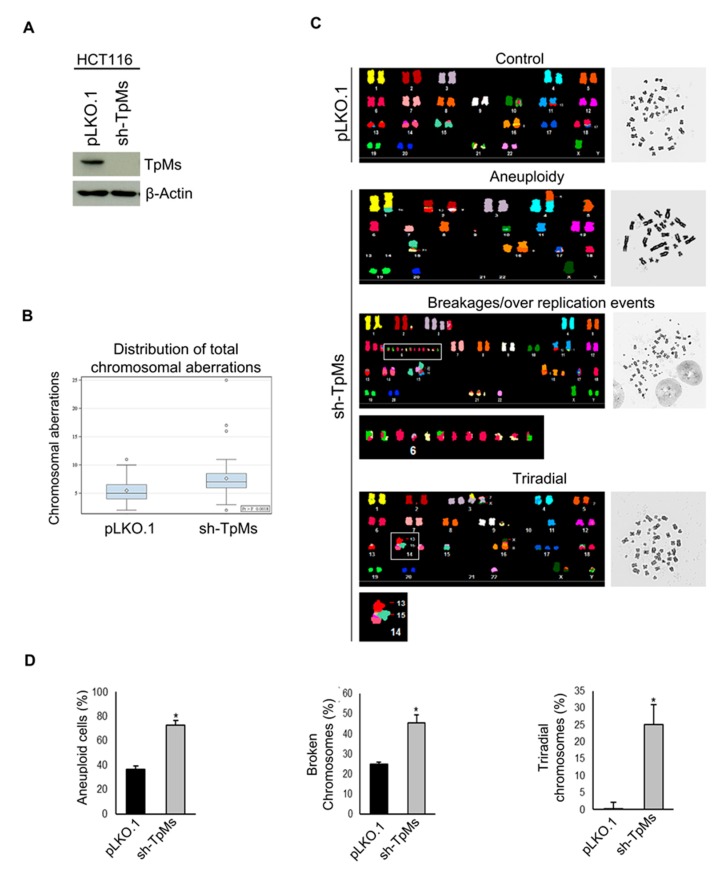
TpMs depletion induces chromosome instability. (**A**) Immunoblot shows TpMs level in HCT116 cells infected with a short hairpin targeting the TpMs coding sequence. (**B**) Box plots show the distribution of total aberrations in HCT116 TpMs-depleted cells compared to control cells (pLKO.1), *p*-value < 0.01. (**C**) Spectral karyotyping (SKY) analysis of TpMs-depleted cells. From the top: HCT116 Karyotype of control cells (pLKO.1); representative karyotypes illustrating an aneuploid cell, broken chromosomes (rectangle) and triradial chromosomes (square) in TpMs-depleted cells (sh-TpMs). (**D**) Quantifications of each type of chromosome aberrations based on the analysis of 44 metaphases (*n* = 44) (*p*-value = 0.0005; *p*-value = 0.0066; *p*-value = 0.0061). Statistical analysis is performed using ANOVA test. Error bars represent s.d. (*n* = 3).

As chromosome mis-segregation may result in chromosomal instability, we performed a detailed analysis of chromosomal aberrations in HCT116 cells, which are known to be endowed with a relatively stable karyotype [16], in which TpMs expression has been silenced by RNAi. TpMs-depleted cells were generated by a retroviral transduction of short hairpins targeting the TpMs coding sequence, as described in the Material and Methods section (Figure 2A, Figure S4). We utilized Spectral Karyotyping (SKY), a fluorescent (multicolor) in situ hybridization (FISH) technique [17]. SKY analysis of 44 metaphase spreads of sh-TpMs HCT116 cells and relative control cells (pLKO.1) showed a near diploid karyotype (2n = 45) and three conserved rearrangements involving chromosome 11 [der(11)t (11;13)], chromosome 16 [der(16)t(8;16)] and chromosome 18 [der(18)t(17;18)]. These characteristics are in agreement with those presented by Karpf et al. in similar experimental conditions (Figure 2C) [18]. Compared to pLKO.1 cells, Sh-TpMs cells exhibit a significant increase in total aberrations (*p* = 0.0007) including structural aberrations as translocations, fusions, insertions, duplications, di-centric chromosomes, tri-radial chromosomes (*p* = 0.0061) and broken chromosomes (*p* = 0.0066) (Figure 2B). Tri-radial chromosomes appear as chromosomes fused together at their centromeres, causing non-disjunction of chromosomes. Additionally, we found numerical defects (aneuploidy) for about 75% of sh-TpMs cells analyzed (2n ≠ 45), compared to 35% of pLKO.1 cells (*p* = 0.0005) (Figure 2C,D). These data demonstrate that TpMs depletion leads to CIN.

### 2.3. TpMs Depletion Induces DNA Damage

**Figure 3:**
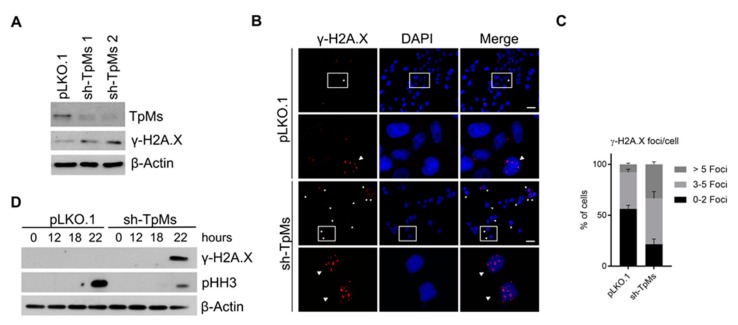
TpMs depletion induces DNA damage. (**A**) γ-H2A.X protein level increases in HCT116 cells stably transfected with lentiviral particles harboring TpMs shRNA (Sh-TpMs) and control shRNA (pLKO.1). Sh-TpMs 1 and Sh-TpMs 2 indicate two different shRNA targeting TpMs. β-Actin protein is used as housekeeping gene. (**B**) Immunofluorescence staining for anti γ-H2A.X antibody in HCT116 cells. (**C**) Quantification of positive cells (%) for γ-H2A.X (γ-H2A.X foci/cell) (*p*-value < 0.0001). Images are obtained by 10× objective and 40× objective for the high magnification. Scale bar 20 µm. (**D**) γ-H2A.X levels analyzed by immunoblot in presence of activated SAC (by nocodazole treatment) shows increased DNA damage in TpMs-depleted HeLa cells, compared to control cells (pLKO.1). Phospho-histone H3 (pHH3) is used as a mitotic marker and β-Actin as a housekeeping gene, *p*-value < 0.0001. Error bars represent s.d. (*n* = 3).

Increasing evidence showed that errors in mitosis such as chromosome mis-segregation can promote chromosome breaks and DNA damage [2]. Therefore, we assessed the presence of DNA damage after TpMs depletion in exponentially growing HCT116 cells in the absence of any spindle inhibitory treatments. Immunoblotting analysis shows that TpMs depletion triggers activation of the DNA double strand breaks (DSBs) marker γ-H2A.X (Figure 3A, Figure S5) [19]. Similar results were observed when γ-H2A.X was visualised as discrete foci by immunofluorescence (Figure 3B,C). Notably, the γ-H2A.X level drastically increases in TpMs-depleted cells subjected to nocodazole treatment for 22 h, while it is absent in nocodazole-treated pLKO.1 control cells (Figure 3D, Figure S6). Taken together, our results indicate that the silencing of TpMs expression leads to chromosome mis-segregation, structural/numerical chromosomes aberrations (Figure 1 and Figure 2) as well as DNA damage (Figure 3).

### 2.4. TpMs Depletion Leads to Reduced Expression Level of Mad2 during Mitosis 

**Figure 4:**
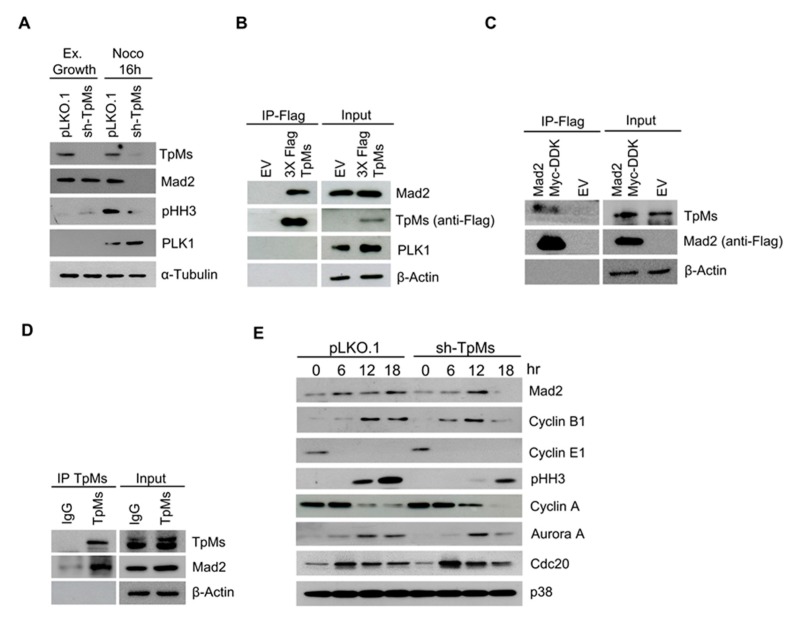
TpMs depletion leads to reduced expression level of Mad2 protein during mitosis. (**A**) HCT116 cells were arrested in mitosis by nocodazole for 16 h; Mad2 levels are revealed by immunoblot. (**B**) HEK293T cells were transfected with empty vector (EV) or Flag-tagged full-length TpMs vector (3X-Flag TpMs). Whole-cell extracts (Input) were immunoprecipitated (IP-FLAG) with anti-FLAG resin. Immunocomplexes are probed with antibodies specific to the indicated proteins. (**C**) HEK293T are transfected with EV or flag-tagged full-length Mad2 (Myc-DDK-Mad2). Whole-cell extracts (Input) were immunoprecipitated (IP-FLAG) with anti-FLAG resin. Immunocomplexes are probed with antibodies specific to the indicated proteins. (**D**) Whole-cell extracts from HEK293T cells were immunoprecipitated with an anti-TpMs antibody. TpMs immunocomplexes were then probed with a Mad2 antibody. (**E**) HeLa cells are synchronized in aphidicolin for 24 h and released in nocodazole. Cells were harvested at different times, and the indicated proteins were detected by immunoblot.

The spindle assembly checkpoint (SAC) prevents chromosome mis-segregation and aneuploidy by delaying sister chromatid separation until all chromosomes have achieved bipolar kinetochore–microtubule attachment [20]. This delay is obtained by inhibiting a complex of specific proteins (anaphase-promoting complex/cyclosome, APC/C) through the activity of spindle checkpoint proteins such as Mad1, Mad2, BubR1, which are recruited to unattached kinetochores [21]. In particular, Mad2 appears to be the critical checkpoint effector of mitosis. Mad2-lacking cells present severely compromised SAC [22]. The single allele deletion of the Mad2 gene results in defective mitotic checkpoint in HCT116 cells and mouse embryonic fibroblasts [23]. To investigate the molecular mechanisms by which TpMs depletion affects the activation of the spindle checkpoint, we assessed the expression of several mitotic spindle checkpoint proteins in TpMs-depleted cells. Our results show that, in response to nocodazole treatment, Mad2 levels are markedly reduced in TpMs-depleted HCT116 cells in comparison with the ones in control cells (Figure 4A, Figure S7). However, Mad2 levels are not reduced in exponentially growing TpMs-depleted cells (mostly in interphase) (Figure 4A, Figure S7), implying that TpMs depletion affects Mad2 level in a cell-cycle dependent manner. Similar results were obtained in HeLa cells (Figures S8A and S9). It is worthwhile noticing that cells ectopically expressing TpMs full-length exhibited higher Mad2 levels when compared with the ones in control cells (Figures S8B and S10). Next, we tested whether TpMs interacts with Mad2 in cultured cells. As shown in Figure 4B and Figure S11, Flag-tagged TpMs in HEK293T cells were found to interact with endogenous Mad2 by coimmunoprecipitation. The interaction between TpMs and Flag-tagged Mad2 by IP following by TpMs immunoblot assay is shown in Figure 4C and Figure S12. The interaction between endogenous TpMs and endogenous Mad2 was also observed (Figure 4D, Figure S13). We also analyzed the expression level of Cyclin B1 in TpMs-depleted cells. Cyclin B1, a regulatory subunit of cyclin-dependent kinase 1 (CDK1), is degraded via the anaphase promoting complex/cyclosome (APC/C). Cyclin B1 is mostly regulated by the SAC as activated SAC prevents Cyclin B1 degradation and delays mitotic exit. Here, we analyzed the level of Cyclin B1 in TpMs-depleted HeLa cells and control cells (pLKO.1). Cells were harvested at different time points during an 18-h time interval after they had been released from the G1/S block into a nocodazole-containing medium (that induces SAC activation). Interestingly, Cyclin B1 was prematurely degraded in TpMs-depleted cells, while it was still present in pLKO.1 control cells (Figure 4E, Figure S14). Thus, the early degradation of Cyclin B1 follows Mad2 degradation when SAC is activated by nocodazole treatment in TpMs-depleted cells. The G2/M marker phospho-histone H3 (pHH3) as well as Aurora A, which is a critical protein for the proper formation of mitotic spindle, were reduced in TpMs-depleted cells in comparison with pLKO.1 control cells (Figure 4E, Figure S14). TpMs appears to be required for the optimal activation of the spindle checkpoint, and, as a consequence, for the fidelity of the mitosis process.

## 3. Discussion

Both in vitro and in vivo observations demonstrate that TpMs protein plays diverse functions in eukaryotic cells that depend on its intracellular localization, ranging from the activity of anchoring microtubules to the centrosome [15] to the negative regulation on primary cilia assembly in G1 phase [14], and finally to interaction between autophagy and endothelial cells [12]. TpMs acts as a tumor suppressor gene being markedly down-regulated in advanced stages of mammary and bladder carcinomas [10,13], and prostate tumors [13]. Many efforts are aiming to investigate the roles of TpMs protein in tumor onset and progression. The present study highlights possible novel roles for TpMs, both as a mitotic checkpoint regulator and a guardian of chromosomal stability.

Our investigations on the cell cycle progression of TpMs-depleted cells through mitosis showed an increased percentage of anaphase chromosome bridges and lagging chromosomes, in comparison with control cells. Anaphase bridges can result from end-to-end chromosome fusion. However, triradial and quadriradial chromosomes can also form such anaphase bridges [24]. Such fusions generate dicentrics, which break apart at the telophase, leaving one daughter cell with an unbalanced translocation and the other one with a terminal deletion. Both daughter cells remain with double strand breaks at the respective chromosomal ends. The latter will continue breakage–bridge–fusion cycles until the double strand break is resolved.

Lagging chromosomes derived from unattached or mis-attached chromosomes, as well as from broken chromosomes lacking centromeric regions and kinetochore structures, produce structural alterations in cancer cells that result in whole chromosome gains and losses [25]. Lagging chromosomes can mis-segregate, leading to aneuploidy (i.e., numerical instability) and to translocations (i.e., structural instabilities) or can segregate as a form sequestered in micronuclei, which still results in effective aneuploidy [3]. A novel form of genetic instability known as “chromothripsis” is thought to be due, in part, to lagging chromosome pulverization [26,27]. The consideration that chromosome aberrations observed in TpMs-depleted cells are typical hallmarks of premature sister–chromatid separation and chromosomal end-to-end fusions, both linked to chromosome mis-segregation, prompted us to deeply analyze chromosome aberrations in metaphase chromosomes of TpMs cells by Spectral Karyotyping (SKY). The analysis revealed various types of defects including premature anaphase onset, followed by numerical (i.e., aneuploidy) and structural chromosome aberrations (i.e., translocations, fusions, insertions, duplications, di-centric chromosomes, tri-radial chromosomes and broken chromosomes). All the above aberrations can cause CIN.

Spectral karyotyping (SKY) reveals chromosomal aberrations that are consistent with telomere dysfunction: for example, the presence of dicentric chromosomes is one of the hallmarks of telomere dysfunction. In this scenario, the telomeres may have been critically shortened and/or uncapped, which enables end-to-end telomeric fusions that may happen between non-homologous or homologous chromosome pairs. End-to-end fusion chromosomes lead to breakage–fusion–bridge (B/F/B) cycles that increase the genetic diversity between tumor cells [28,29].

Activation of the H2AX Histone marker revealed the presence of DNA double strand breaks (DSBs) in TpMs-depleted cells, either when the cells are exponentially growing or when cells are synchronized by aphidicolin and arrested in mitosis by nocodazole-treatment. According to Janssen, A. et al. [2], we observed that the DNA damage occurring in TpMs-depleted cells that are committed to mitosis might result from defective chromosomes segregation that, in turn, is consistent with defective SAC activity. 

To date, DNA damage response (DDR) has been unanimously believed to safeguard the cell genome by acting in the interphase of the cell cycle. Orthwein et al. [30] showed that major DSBs repair processes are shut down during mitosis because DSBs repair performed during mitosis can destabilize the genome and result in deleterious effects. In accordance, DNA damage response (DDR) protects genome stability by acting during the interphase of the cell cycle. Recently, it has been demonstrated that DDR is also involved in chromosome segregation during mitosis progression in normal cells (i.e., mitotic DDR) [3]. In addition, a huge array of data point out that cancer cells may acquire the ability of distorting the protective role of mitotic DDR. The DNA damage caused by chromosome mis-segregation during the mitosis of cancer cells can introduce numerical as well as structural alterations in chromosomes. For yet unknown reasons, these alterations may trigger a defective process of mitotic DDR that propagates the genomic instability of cancer cells. This process induces chromosome mis-segregation by linking structural and numerical alterations in chromosomes that were traditionally thought to occur independently [3,31]. Understanding the mechanisms through which cancer cells coopt and distort mitotic DDR to propagate their chromosomal instabilities (CIN) is of crucial importance [3].

Chromosome mis-segregation and aneuploidy are generally prevented by the activity of the SAC that delays sister chromatid separation until all chromosomes have achieved bipolar kinetochore–microtubule attachment [20]. Mad2 protein is a central component of the SAC and acts as the critical spindle checkpoint effector of mitosis. The canonical role of Mad2 is to detect unattached kinetochores in order to assure accurate chromosome segregation. In response to microtubule disruption, Mad2, together with other checkpoint proteins, transmits a “wait” signal to prevent anaphase entry and chromosome segregation through inhibition of the anaphase-promoting complex/cyclosome by interacting with Cdc20 [21]. Mad2-lacking cells present severely compromised SAC [22]. The deletion of a single allele of Mad2 gene results in a defective mitotic checkpoint in HCT116 cells and mouse embryonic fibroblasts [23]. In some organisms, Mad2 exhibits roles in preventing mutations/recombination through the DDR [32]. We investigated whether DNA damage and chromosomal aberrations in TpMs-depleted cells might be consistent with defective SAC by assessing the expression level of Mad2 and several checkpoint proteins of the mitotic spindle. Mad2 showed a significant decreased level in TpMs-depleted cells via a cell-cycle-dependent manner, thus suggesting that TpMs function(s) in the SAC is (are) associated to or mediated by Mad2. Impaired Mad2 levels are known to cause premature activation of the APC/C, which usually allows the proper anaphase onset. It is conceivable that the decreased Mad2 level in TpMs-depleted cells fails to inhibit APC/C activity causing the anaphase to occur at earlier times than in the typical cell cycle progressing [33]. Moreover, we reported the early degradation of Cyclin B1 in TpMs-deprived cells. Cyclin B1 is a regulatory subunit of cyclin-dependent kinase 1 (CDK1) that delays the cells’ exit from the mitotic phase, being degraded by APC/C [33]. We point out that the early decrease in Cyclin B1 in TpMs-deprived cells is consistent with the increased rate of mitotic slippage revealed in these cells. This finding suggests that TpMs-deprived cells escape from the mitotic checkpoint, and supports the hypothesis of TpMs involvement in mitosis progression and spindle checkpoint activation. The defects we reported in TpMs-depleted cells, like failure in chromosome segregation and premature anaphase onset, are similar to the results obtained by Meraldi, P. et al. [34] upon the cell depletion of spindle checkpoint components Mad2 or BubR1 (in Mad2 or BubR1-depleted cells). It is believed that Mad2 may reach the spindle poles before anaphase onset through the microtubule-mediated transit between kinetochores and spindle poles [35]. Mad2 depletion may also result in a premature anaphase onset, which is kinetochore-independent. Differently, knockdown of other spindle checkpoint components, such as Bub3 and Bub1, impairs only kinetochore-dependent checkpoint functions [34]. Based on our data, we hypothesize a dynamic interaction between Mad2 and TpMs that affects the functioning of the complex constituted by the spindle poles–centrosome and kinetochore. Mad2 and TpMs interaction might participate in controlling the activity of the SAC process.

In future studies that build on this current work, we will investigate the possibility that the in vitro evidence of TpMs and Mad2 interaction may be utilized for the development of new therapeutic interventions in cancer therapy. The TpMs gene has been identified as a possible candidate tumor suppressor gene because it frequently undergoes homozygous deletion or mutation in various cell lines derived from human cancer and solid human tumors. When transiently over-expressed, TpMs inhibits colony formation, cell growth, migration, invasion, adhesion in vitro and tumor formation in vivo, while it is pro-apoptotic. All these features are commonly shared by established tumor suppressor genes. TpMs expression was reduced in bioptic specimens of cancers in comparison with the health counterpart in 22% of advanced bladder cancer, in 23% of breast carcinomas and in 35% of prostate cancers that we analyzed [10,13].

The chromosome aberrations detected in TpMs-depleted cells represent, at the moment, the unique connection we identified between the tumor suppression ability of TpMs and its function in maintaining chromosomal segregation. This connection suggests that the reduced TpMs level detected in human biopsies of various cancer types might cause chromosome aberrations in these cancers, similar to the ones we revealed to occur in vitro in cancer-derived cells. In vitro findings on TpMs and Mad2 suggest the need to extend the determination of TpMs levels to a major number of diverse cancer types accompanied by the determination of Mad2 level [32,36], with the purpose of identifying diverse patient phenotypes related to TpMs and Mad2 expression. We speculate that the level of TpMs and Mad2 proteins might be exploited as possible relevant biomarkers that are useful to obtain therapeutic interventions for patients bearing solid cancer. These patients might be identified, selected and stratified in different groups based on the TpMs and Mad2 levels of their biopsies, relative to the level of healthy counterparts. The patients of each group are presumed to be differently susceptible to chromosomal mis-segregation and/or persistent DNA damage. Thus, patients could be treated with therapeutic interventions [37,38,39,40] that target their individual phenotype with the aim of possibly improving their clinical outcome.

## 4. Materials and Methods

### 4.1. Cell Culture and Reagents

HeLa, HCT116, HEK293T cell lines were grown in DMEM-high Glucose medium, supplemented with 10% fetal bovine serum (FBS), HUVEC cell lines were grown in F-12K medium with supplements. All cell lines were maintained in the incubator in a humidified atmosphere containing 5% CO2 at 37 °C. All cell lines were obtained from ATCC and European collection of cell cultures, ECACC via Sigma. All studies were performed in Mycoplasma negative cells, as routinely determined with the MycoAlert Mycoplasma detection kit (Lonza, Walkersville, MD, USA). Reagents: Aphidicolin (Sigma Aldrich A078, 2 µg/mL); Nocodazole (Sigma Aldrich M1404, 0.1 µg/mL); monastrol (Cayman Chemical 15044, Ann Arbor, MI, USA); MG132 (Sigma Aldrich M8699-1MG); Propidium Iodide (50 µg/mL; Sigma Aldrich, St. Louis, MO, USA); Puromycin (Thermo Fisher Scientific A1113803, 1 μg/mL); Lipofectamine RNAiMax reagent (Invitrogen, Carlsbad, CA, USA).

### 4.2. Lentivirus and Retrovirus Production, Plasmid Constructs and siRNAs

The pLKO.1 DNA plasmids containing the shRNA sequence against human TPMS (TCHP) was purchased from Sigma Aldrich (Mission^®^RNAi TRCN0000127662 and TRCN TRCN0000130868). The scrambled sequence shRNA plasmid was purchased from Addgene, plasmid #1864. The packaging plasmids used were pCMV-ΔR8.2Δvpr, plasmid #8455 and pCMV-VSVG, plasmid #8454 from Addgene. Retroviral particles were generated by transient transfection of the vector-histone H2B into phoenix-ampho cells by using calcium phosphate method. The resulting supernatant was used to infect Hela and HCT116 cells. To generate an expression plasmid for 3xFLAG–tagged, the full-length TpMs coding sequence was amplified by PCR with primers having the sequence 5′-GATGACAAGCTTGGAAACTCCGAGCCTCAGAGA-3′ and 5′ GGATCCTCTAGATTCTCTGTACTTATGGTACCC-3′. The PCR product was digested with HindIII and XbaI, and the resulting DNA fragment was inserted into p3xFLAG-CMV-7.1 (Sigma Aldrich) to prepare p3xFLAG-TCHP. A 3xFLAG–TPMS (TCHP) coding sequence was then amplified by PCR, the products were purified by gel and verified by sequencing. siRNA for TpMs are SMARTpool: ON-TARGETplus TCHP siRNA (Dharmacon).

### 4.3. Live Cell Imaging

Cells expressing GFP-histone H2B (H2B-GFP) were grown in glass bottom dishes and transfected with siRNA-TpMs and siRNA-CTRL. The cells were synchronized by aphidicolin for 24 h and released in DMEM medium. Cells were imaged for 24 h by TCS Leica SP8 microscope. The movies were generated using the ImageJ software (NIH).

### 4.4. Spectral Karyotyping

The HCT116 cells were treated with nocodazole (1 µg/mL) for 2 h and after analyzed by Spectral karyotyping analysis (SKY). SKY was performed by using the SKY kit for human chromosomes (ASI, Vista, CA, USA) and following the manufacturer’s protocol. Slides were imaged by using an Axioplan 2 microscope with a 63/1.4 oil objective (Zeiss, Toronto, ON, Canada) and were analyzed by using Case Data Manager 4.0 software (ASI, Vista, CA, USA). A total of 44 metaphases were imaged and analyzed for each sample. Rearrangements were scored and statistically analyzed.

### 4.5. Immunofluorescence

The cells were grown in chamber slides to 1 × 10^6^ cell/mL and fixed with a solution of 95% ethanol and 5% acetic acid for 5 min. The blocking phase was performed in 3% BSA/TBS for 30 min. Cells were incubated with the primary antibody anti-phospho-Histone h2A.X (Ser 139) for 1 h and then with the secondary antibody of goat anti-mouse AlexaFluor 594 (Molecular Probes, Invitrogen). Cells washes were performed with Tris Buffered Saline (TBS). Images were acquired with Carl Zeiss Microscope AxioSkop 40 FL. HUVECs cells were plated on fibronectin-coated glass coverslips. Twenty-four hours later, the slides were fixed with 4% paraformaldehyde, permeabilized with 0.05% Triton X-100 in PBS, then incubated with the primary antibody (1:400) in 3% BSA overnight at 4 ℃. Secondary antibodies diluted 1:1000 in 3% BSA. Slides were imaged on Zeiss LSM-780 confocal. Primary and secondary antibodies used for immunofluorescence were: αTubulin (Abcam, ab184613); Alexa Fluor^®^ Plus 488 (ThermoFisher A32731, Rockford, IL, USA).

### 4.6. Immunoblotting

Cells were harvested from the substrate by scraping or by utilizing trypsin solution and pelleted before being lysed. Lysis of the cells was performed on ice in RIPA buffer (Cell signaling #9806) containing 1mM PMSF (Cell Signalling #8553). Cells proteins were quantified by using the Bradford assay (Sigma Aldrich B6913). Equal amounts of proteins were loaded onto SDS-Polyacrylamide gels and transferred to PVDF membrane. Protein separation in the membranes was then blocked with 5% non-fat milk in TBST 0.1%. Immunoblotting of the proteins was performed overnight at 4 °C with the following primary antibodies: anti-TpMs (Sigma ATLAS HPA038638 and Santa Cruz sc515025); anti-phospho-histoneH3 (EMD Millipore); anti-phospho-histone H2A.X Ser139 (EMD Millipore 05-636); anti-ARK1 (Aurora A) (Santa Cruz Biotechnology); anti-cyclinB1 (Bethy Laboratories A305-000A); anti-Mad2 (Bethy Laboratories A300-301A); cyclin A (Thermo Fisher); Cdc20 (Bethy Laboratories A301-180A); anti-M2 FLAG (Sigma Aldrich F1804); p38 (Santa Cruz Biotechnology sc-81621); anti-β-Actin (Millipore MAB1501). Horseradish peroxidase (HRP)-conjugated anti-mouse or anti-rabbit (Sigma-Adrich A5906, A0545) secondary IgG antibodies were used. The signal of immunoblotted proteins was visualized by using the ECL Plus Western Blotting Detection System (GE Healthcare Biosciences, Freiburg, Germany). Pixel intensity/quantification was performed using ImageJ.

### 4.7. Immunoprecipitation

Cells were washed and collected in ice-cold PBS and then lysed (30 min in ice) by a buffer containing 40 mM HEPES pH 7.4, 5 mM MgCl2, 0.2% NP-40, 10 mM NaF and protease and phosphatase inhibitors. Cells extracts were obtained by centrifugation of the lysates (20 min at 4 °C) and filtered with a 0.45 µm filter. Then, cell extracts were incubated (overnight at 4 °C) with EZview™ Red ANTI-FLAG^®^ M2 Affinity Gel (Sigma Aldrich F2426) and washed with a lysis buffer containing 150 mM NaCl. For the endogenous TpMs immunoprecipitation, the cell extracts were first precleared by incubation with protein A-Sepharose beads (Thermo Fisher Scientific, Camarillo, CA, USA) for 45 min at 4 °C. Precleared extracts were incubated with the primary antibody for 3 h at 4 °C, and then with protein A-Sepharose beads for 45 min. After the beads were washed four times with a lysis buffer, the proteins were eluted in 5× Laemmli sample buffer (50 mM Tris-HCl, pH 6.8, 2% (*w/v*) SDS, 5% (*v/v*), β-mercaptoethanol, 0.1% (*w/v*) bromophenolblue and 1% (*v/v*) glycerol). For immunoblotting, proteins were separated by SDS polyacrylamide gel electrophoresis (SDS-PAGE), transferred onto PVDF membranes (GE Healthcare Biosciences), and incubated with the indicated antibodies.

### 4.8. Statistical Analysis

All data were obtained by the mean of three experiments. Statistical analysis was performed by using an Unpaired *t*-Test or by using a Wilcoxon TEST (Rank Sums) and ANOVA. *p*-value of <0.01 was considered significant. Analyses were performed using GraphPad Prism v7 (La Jolla, CA, USA).

## 5. Conclusions

In conclusion, we demonstrated that TpMs might play a role in guarding mitosis fidelity and CIN by enabling optimal activation of the SAC. TpMs depletion leads to chromosome mis-segregation followed by a persistent level of DNA damage and numerical/structural chromosome instabilities. These features might be exploited for therapeutic implications.

## Supplementary Materials

The following are available online at https://www.mdpi.com/2072-6694/12/4/993/s1, Figure S1: Immunoblot showing decreased levels of TpMs after transient transfection with siRNA-CTRL and siRNA-TpMs in HCT116 cells stably expressing enhanced green fluorescent protein-labelled histone H2B, Figure S2: TpMs-depleted cells display defects of the mitotic checkpoint and a faster exit from mitosis, Figure S3: Immunoblot showing the p21 protein levels in HeLa cells. Cells were harvested at different time points during a 18-h time interval after they had been released from G1/S block into nocodazole-containing medium, Figure S4: Immunoblot shows TpMs level in HCT116 cells infected with a short hairpin targeting the TpMs coding sequence, Figure S5: γ-H2A.X protein level increases in HCT116 cells stably transfected with lentiviral particles harboring TpMs shRNA (Sh-TpMs) and control shRNA (pLKO.1). Sh-TpMs 1 and Sh-TpMs 2 indicate two different shRNA targeting TpMs. β-Actin protein is used as housekeeping gene, Figure S6: γ-H2A.X levels analyzed by immunoblot in presence of activated SAC (by nocodazole treatment) shows increased DNA damage in TpMs-depleted HeLa cells, compared to control cells (pLKO.1). Phospho-histone H3 (pHH3) is used as mitotic marker and p38 as housekeeping gene, Figure S7: HCT116 cells were arrested in mitosis by nocodazole for 16 h; Mad2 levels are revealed by immunoblot, Figure S8: TpMs expression modulates Mad2 protein levels, Figure S9: TpMs was silenced by a short harpin RNA targeting TpMs (sh-TpMs). HeLa cells were arrested in mitosis by nocodazole treatment for 16 h. Cells were collected and lysed. Mad2 levels were detected by immunoblotting, Figure S10: Ectopic expression of TpMs leads to increased Mad2 levels. HEK293T cells were transiently transfected with empty vector (EV) or Flag-tagged full-length TpMs vector (3X-Flag-TpMs). Mad2 levels were evaluated by immunoblotting, Figure S11: HEK293T cells were transfected with empty vector (EV) or Flag-tagged full-length TpMs vector (3X-Flag TpMs). Whole-cell extracts (Input) were immunoprecipitated (IP-FLAG) with anti-FLAG resin. Immunocomplexes are probed with antibodies specific for the indicated proteins, Figure S12: HEK293T are transfected with EV or flag-tagged full-length Mad2 (Myc-DDK-Mad2). Whole-cell extracts (Input) were immunoprecipitated (IP-FLAG) with anti-FLAG resin. Immunocomplexes are probed with antibodies specific for the indicated proteins, Figure S13: Whole-cell extracts from HEK293T cells were immunoprecipitated with an anti-TpMs antibody. TpMs immunocomplexes were then probed with Mad2 antibody, Figure S14: HeLa cells are synchronized in aphidicolin for 24 h and released in nocodazole. Cells were harvested at different times, and the indicated proteins were detected by immunoblot, Video S1: siRNA-CTRL, Video S2: siRNA-TpMs-bridge, Video S3: siRNA-TpMs-lagging.
